# Solid-binding peptides for immobilisation of thermostable enzymes to hydrolyse biomass polysaccharides

**DOI:** 10.1186/s13068-017-0715-2

**Published:** 2017-02-02

**Authors:** Andrew Care, Kerstin Petroll, Emily S. Y. Gibson, Peter L. Bergquist, Anwar Sunna

**Affiliations:** 1 Department of Chemistry and Biomolecular Sciences, Macquarie University, Sydney, Australia; 2 ARC Centre of Excellence for Nanoscale BioPhotonics (CNBP), Macquarie University, Sydney, Australia; 3 Department of Molecular Medicine & Pathology, Medical School, University of Auckland, Auckland, New Zealand; 4 Biomolecular Discovery and Design Research Centre, Macquarie University, Sydney, Australia

**Keywords:** Solid-binding peptide, Thermostable enzymes, CLEAs, Enzyme immobilisation, Biocatalytic modules

## Abstract

**Background:**

Solid-binding peptides (SBPs) bind strongly to a diverse range of solid materials without the need for any chemical reactions. They have been used mainly for the functionalisation of nanomaterials but little is known about their use for the immobilisation of thermostable enzymes and their feasibility in industrial-scale biocatalysis.

**Results:**

A silica-binding SBP sequence was fused genetically to three thermostable hemicellulases. The resulting enzymes were active after fusion and exhibited identical pH and temperature optima but differing thermostabilities when compared to their corresponding unmodified enzymes. The silica-binding peptide mediated the efficient immobilisation of each enzyme onto zeolite, demonstrating the construction of single enzyme biocatalytic modules. Cross-linked enzyme aggregates (CLEAs) of enzyme preparations either with or without zeolite immobilisation displayed greater activity retention during enzyme recycling than those of free enzymes (without silica-binding peptide) or zeolite-bound enzymes without any crosslinking. CLEA preparations comprising all three enzymes simultaneously immobilised onto zeolite enabled the formation of multiple enzyme biocatalytic modules which were shown to degrade several hemicellulosic substrates.

**Conclusions:**

The current work introduced the construction of functional biocatalytic modules for the hydrolysis of simple and complex polysaccharides. This technology exploited a silica-binding SBP to mediate effectively the rapid and simple immobilisation of thermostable enzymes onto readily-available and inexpensive silica-based matrices. A conceptual application of biocatalytic modules consisting of single or multiple enzymes was validated by hydrolysing various hemicellulosic polysaccharides.

## Background

The immobilisation of enzymes onto solid supports can be achieved using various chemical and physical techniques, such as adsorption, covalent attachment, entrapment/encapsulation, and crosslinking [1]. Compared to soluble enzymes, immobilised enzymes offer enhanced stability and easy removal from a reaction mixture, enabling repetitive use in batch and continuous bioprocesses, rapid termination of reactions, controlled product formation and the provision of flexibility to industrial bioprocesses (e.g. biofuel production) [2, 3]. Unfortunately, conventional enzyme immobilisation methods often result in the non-uniform orientation of enzymes and unwanted conformational changes that distort active sites and thus reduce the catalytic activity of the enzyme [4–7].

Solid-binding peptides (SBPs) exhibit binding affinity and selectivity to the surfaces of solid materials, including support materials used in biocatalysis e.g., silica, zeolite, glass, metals and polymers [8]. They bind strongly to their cognate solids by adopting specific conformations that support numerous non-covalent interactions (e.g. electrostatic bonding) between key amino acids and the solid surface, often resulting in binding affinities (*K*_d_) in the sub-micromolar and nanomolar range [9]. Furthermore, SBPs can be integrated easily into the permissive regions of proteins (e.g. N- or C-terminal) using genetic engineering techniques to produce bifunctional fusion proteins. Consequently, SBPs are employed frequently as molecular linkers and/or anchoring molecules for the immobilisation of functional proteins onto solid surfaces in a single-step and without the need for any complicated chemical reactions or physical treatments [8].

SBPs have been shown to direct the oriented immobilisation of enzymes onto solid supports with retention of native enzyme structure and catalytic activity (e.g. *k*_cat_/Km) [10, 11]. For example, the immobilisation of alkaline phosphatase (AP) onto gold surfaces via a gold-binding peptide (GBP-1) has been reported [12]. When compared to native AP, a GBP1–AP fusion displayed high binding affinity to gold surfaces and self-assembled into densely packed, uniform protein monolayers that exhibited improved enzyme orientation and greater enzymatic activity per unit of area than AP without GBP1. Additionally, biophysical studies have shown that GBP1 is capable of facilitating the immobilisation of enzymes without any observable effect on their native structural conformation [13].

SBPs have been used primarily as molecular tools for the functionalisation of nanomaterials and the practical application of SBP-enzyme fusions are limited currently to chemo/biosensors and bioassays that require exotic and expensive laboratory-based matrices that may not be realistic economically for large-scale biotechnological processes [8, 10, 13]. Silica, zeolite and other mesoporous silica-based materials are used commonly in industry as support matrices in a number of applications and the provision for bulk supply is well established. These inorganic matrices are usually considered the most suitable for the immobilisation of enzymes [14, 15]. They do not require any chemical functionalisation and can be synthesised or mined cheaply. In particular, silica can be tailored easily to provide a large range of porous surfaces, surface functionalities and processing conditions [14]. However, use of unmodified silica as an inorganic support is unusual, as supports are usually functionalised prior to immobilisation of a biomolecule, both in single and multiple enzyme immobilisation [16]. Mesoporous nanomaterials are used because of their high surface area, controlled porosity and simple adsorption and desorption [17]. Silica-based supports show high mechanical strength and microbial resistance compared to other polysaccharide-based adsorbents [15, 18]. These considerations make silica-based materials well-suited for industrial-scale processes. There are few reports regarding inexpensive matrices and little is known about the use of SBPs for the immobilisation of thermophilic enzymes and their feasibility in industrial-scale biocatalysis.

We reported previously the application of a unique SBP of amino acid sequence (VKTQATSREEPPRLPSKHRPG)_4_VKTQTAS (referred to as the linker, L) that binds strongly to a range of inexpensive silica-based materials (e.g. natural or synthetic zeolites and silica) [16, 19–22]. Upon binding to silica surfaces, the linker is thought to undergo conformational changes that promote multiple electrostatic interactions between its many positively-charged residues (K and R) and the negatively-charged silica. Using this technology, a number of linker-fusion proteins were constructed for direct and effective functionalisation of nanomaterial surfaces [16, 19, 21, 22].

Further extensions of this technology to single enzymes and enzyme complexes that can be designed as biocatalytic modules using a silica-binding SBP and low cost bulk silica-based matrices can be envisaged. Single biocatalytic modules consist of one type of enzyme immobilised per matrix molecule, whereas multiple enzyme biocatalytic modules consist of more than one enzyme. Biocatalytic modules can be used to replace industrial chemical catalysts due to their high specificity and high efficiency under mild reaction conditions [17].

Here we focus particularly on the effect that incorporating the silica-binding peptide has on the activity and function of three industrially-relevant thermostable enzymes, providing basic groundwork for future studies on the concept of immobilisation of a biomass degradation pathway consisting of multiple enzymes. β-Glucosidase is the last enzyme in the classical cellulose degradation pathway and unlike the other cellulases, it cannot be recycled by exposure to fresh substrate so it is a priority candidate enzyme for immobilisation [23]. To further complement this work, a thermostable β-mannanase (ManA) and β-xylanase (XynB) from *Dictyoglomus thermophilum* were used to supply additional data on the functionality of the silica-binding peptide. Both enzymes are hemicellulases that catalyse the hydrolysis of the polysaccharides mannan (1,4-linked β-D-mannopyranosyl residues) and xylan (1,4-linked β-d-xylopyranosyl residues), respectively. Hemicellulases, in combination with cellulose-degrading enzymes, play an important role in the efficient degradation of plant biomass.

This study demonstrated the application of the silica-binding SBP-mediated immobilisation of industrially-relevant enzymes onto a bulk and low-cost solid zeolite matrix. Additional introduction of crosslinking into the immobilised enzymes to form single and multiple enzyme biocatalytic modules further highlighted the potential of this platform technology for its translation into industrial-scale processes.

## Results

### Construction of thermophilic genes as linker fusions and their effect on enzyme activity

The linker (L) sequence was incorporated at the N- or C-terminus of three thermostable hemicellulases isolated from thermophilic bacteria (see Table 1). The resulting fusion proteins (L-enzymes) and enzymes (without linker) were expressed in *Escherichia coli* and partially purified by heat treatment as described in Methods.

**Table 1:**
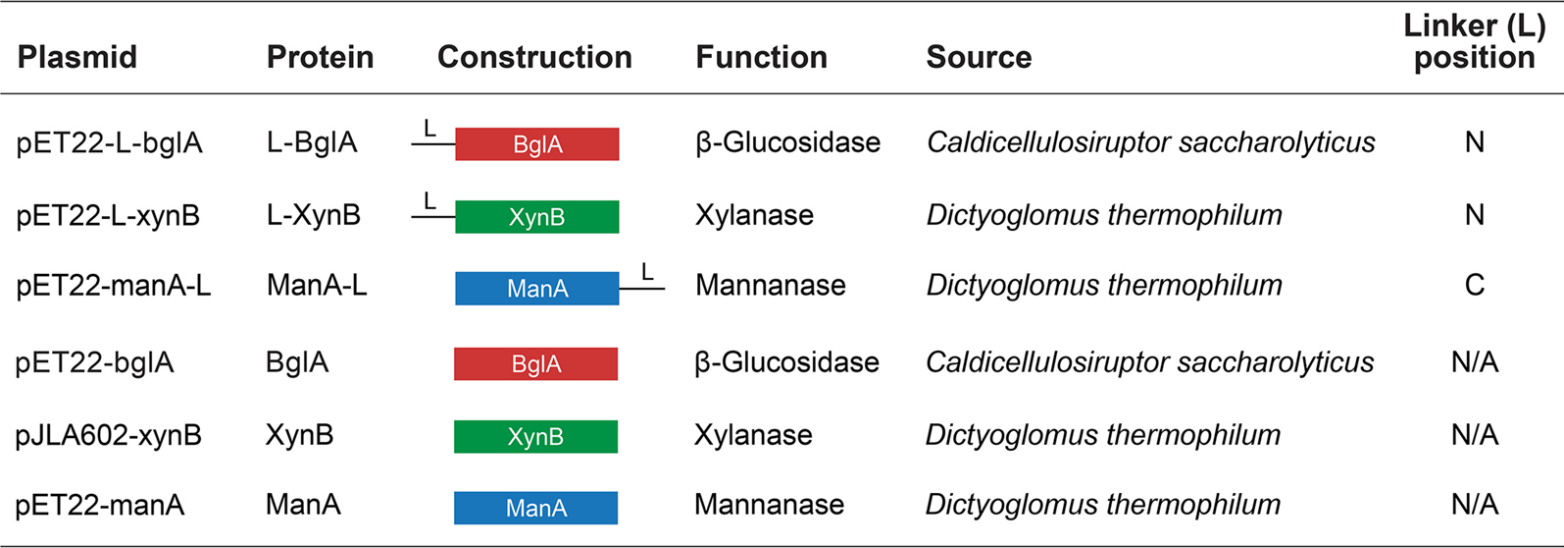
Enzyme constructions used

*N* Linker is at N-terminus of fusion protein, *C* linker is at C-terminus of fusion protein

The optimum pH, temperature and thermostability of each enzyme (with and without linker) was compared to investigate the effect of linker fusion on the operational activity and stability of the enzymes. Table 2 shows that all L-enzymes remained active after fusion and displayed maximal activity at the same pH and temperature optimum as their counterpart enzymes without linker. Thermostability experiments in the absence of substrate showed that L-XynB and ManA-L retained 100% of their enzymatic activity after 6 h incubation at 70 °C. In earlier reports, XynB and ManA (without linker) showed no inactivation following 6 h incubation at 80 °C [24, 25]. However, under similar conditions, L-XynB and ManA-L retained only 73 and 19% of their initial activities, respectively. BglA was the least thermostable enzyme tested as it retained only 69% of its activity after 1 h of incubation at 80 °C whilst L-BglA retained 35%. BglA was reported previously to have lost 50% of its initial activity at 80 °C after 15 and 24 min [26, 27]. Similarly, a recombinantly-expressed and purified β-glucosidase (CbBgl1A) from another *Caldicellulosiruptor* species, *Cs. bescii*, showed a half-life of 20 min at 80 °C [28]. Initially the half-life at 80 °C of the BglA produced in *E. coli* was reported to be 14 h [29].

**Table 2:** Effect of linker fusion on the optimal pH, apparent temperature optima and thermostability of BglA, XynB, and ManA

	BglA	L-BglA	XynB	L-XynB	ManA	ManA-L
Optimum pH	6.0	6.0	6.5	6.5	5.0	5.0
Optimum temperature (°C)	85	85	80	80	85	85
Thermostability at (°C)	Relative activity (%)					
70	81^a^	54^a^	100^b^	100	100^c^	100
80	69^a^	35^a^	100^b^	73	100^c^	19

^a^Samples incubated for 1 h at the specified temperature. All other samples were incubated for 6 h

^b^Morris et al. [24]

^c^Gibbs et al. [25]

### Enzyme immobilisation

#### Zeolite-bound enzymes (zeo-L-enzymes)

**Fig. 1:**
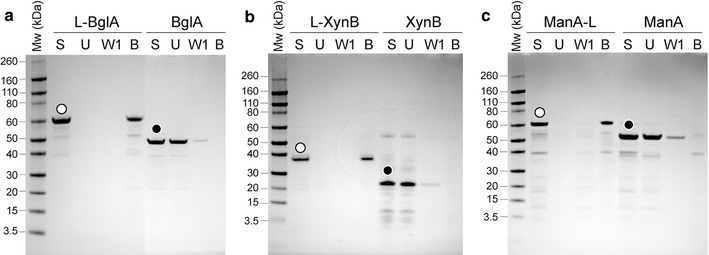
SDS-PAGE analysis showing the zeolite-binding capacity of L-enzymes (*white circle*) and enzymes (without linker) (*black circle*). **a** L-BglA and BglA, **b** L-XynB and XynB, **c** ManA-L and ManA. Standard zeolite binding assays were performed with partially-purified proteins as described in the “Methods” section. *S* starting protein sample, *U* unbound protein fraction, *W1* wash fractions (1 of 3 total), *B* zeolite-bound protein fraction

The linker has been reported to mediate the immobilisation of proteins onto various silica-based materials, including natural and synthetic zeolites [16, 22]. Standard zeolite binding assays were performed with partially purified enzymes (with and without the linker, Fig. 1). All enzymes carrying the linker (L-BglA, L-XynB and ManA-L) displayed strong binding affinity for the zeolite matrix with all of their initial protein present in the final zeolite-bound fraction after a stringent washing procedure. In contrast, the enzymes without linker (BglA, XynB and ManA) did not bind to zeolite and most of their initial protein was observed in the unbound fraction with minimal protein present in initial wash fraction. These results indicated that introduction of the linker sequence into the three thermostable enzymes did not have any negative effects on the zeolite binding affinity of the linker. Furthermore, the position of the linker (N- or C-terminus) with respect to the enzyme sequence did not affect its zeolite binding function.

**Fig. 2:**
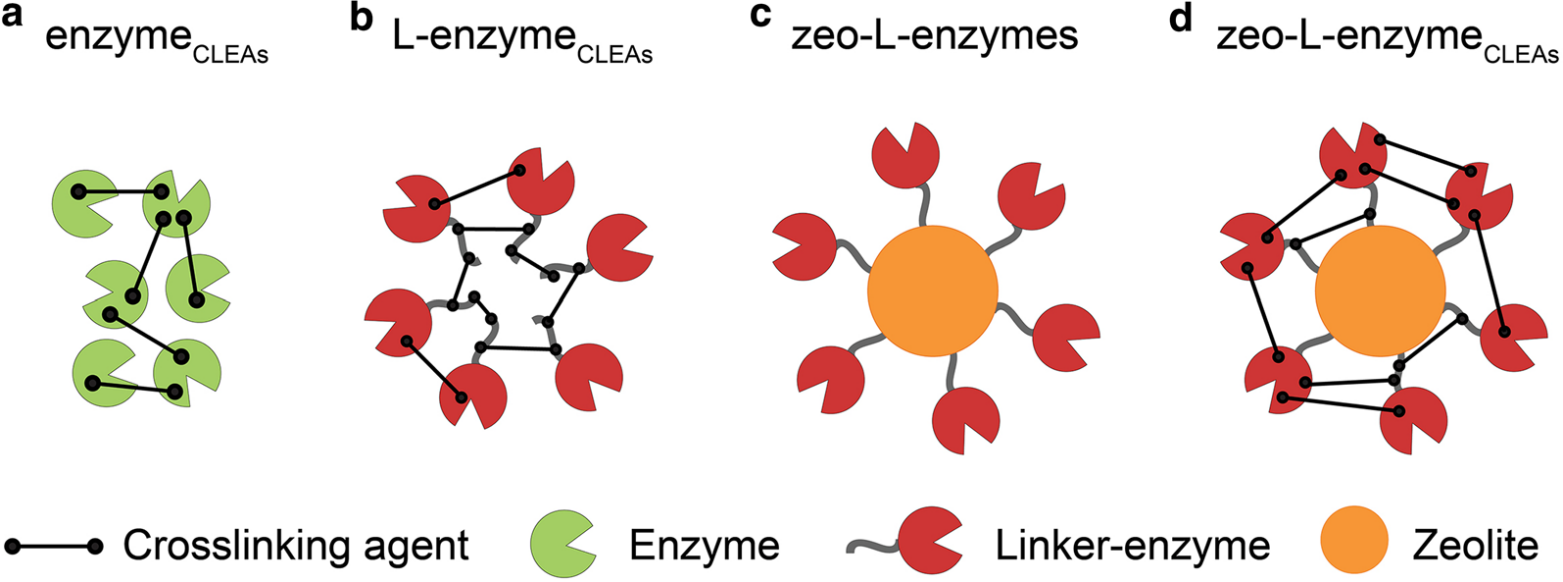
Diagrammatic representation showing the various forms of immobilised thermostable enzymes prepared in this study. **a** enzyme_CLEAs_, CLEAs of enzymes (without the linker); **b** L-enzyme_CLEAs_, CLEAs of L-enzymes; **c** zeo-L-enzymes, zeolite-bound L-enzymes; **d** zeo-L-enzyme_CLEAs_, CLEAs of zeolite-bound L enzymes

Samples containing the enzymes bound to zeolite via the linker (zeo-L-enzymes) (Fig. 2c) were prepared also using the standard binding assays of partially purified L-enzymes without applying the final SDS heat elution step which normally elutes the L-enzyme from the matrix. All enzymes carrying the linker (L-BglA, L-XynB and ManA-L) displayed strong binding affinity for the zeolite matrix and their zeolite-bound fractions and like the free enzymes, they retained their enzymatic activity (data not shown). Enzymes without the linker (BglA, XynB and ManA) displayed no binding affinity to the zeolite matrix and accordingly, almost all of their respective enzymatic activities were found in the unbound fractions with minimal activity present in the wash fractions.

#### CLEAs of free enzymes (enzyme_CLEAs_)

Crosslinked enzyme aggregates (CLEAs) are considered to be an effective carrier-free enzyme immobilisation strategy [30, 31]. CLEAs are prepared by aggregating enzymes by the addition of common protein precipitants (e.g. ammonium sulphate, polyethylene glycol and ethanol). A subsequent crosslinking step assures that the insolubility and pre-organised structure of the aggregate is maintained as well as the catalytic activity of the aggregated enzyme. CLEAs typically display improved storage and operational stability and are easy to recover and recycle.

CLEAs of free enzymes (with and without linker) were prepared by ethanol precipitation and subsequent crosslinking with glutaraldehyde at 4 °C (Fig. 2a, b). Several protein precipitants were tested, including ammonium sulfate and isopropanol, but ethanol provided the most rapid and complete precipitation. Allowing the crosslinking reaction to occur overnight without shaking gave the best results, resulting in low enzyme activity in the supernatant and high activity for the resulting CLEAs.

#### CLEAs of zeolite-bound enzymes (zeo-L-enzyme_CLEAs_)

**Fig. 3:**
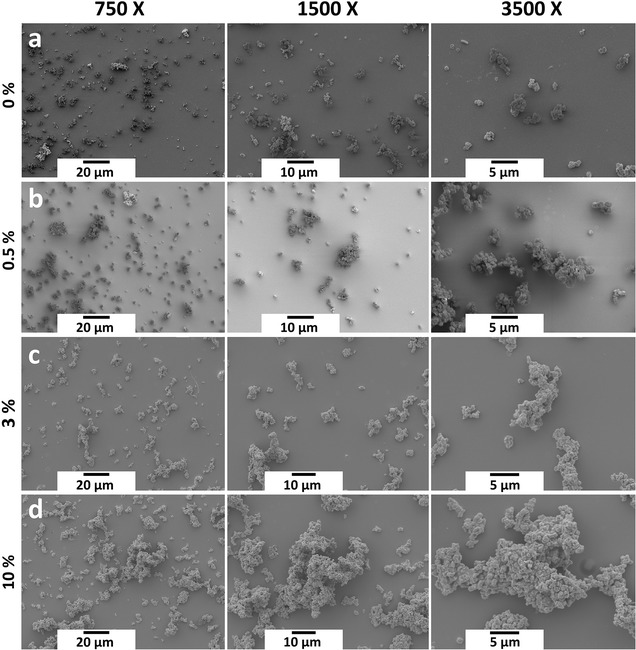
SEM and FESEM images (750×, 1500×, and 3500× magnification) of zeo-L-BglA_CLEAs_ prepared with different concentrations of glutaraldehyde crosslinker. **a** non-crosslinked control, zeo-L-BglA, **b** 0.5% glutaraldehyde zeo-L-BglA_CLEAs_, **c** 3% glutaraldehyde zeo-L-BglA_CLEAs_, **d** 10% glutaraldehyde zeo-L-BglA_CLEAs_. The **b** series of images were taken using a FESEM, which can operate at lower voltages than a standard SEM to reduce charging and image artefacts that occur when negative charges build up on a specimen. This phenomenon occurs in specimens that are not electrically grounded properly, as observed with the smaller particles

Immobilisation onto solid supports imparts mechanical and operational stability to CLEAs and allows their separation from the reaction mixture [32]. The possibility that zeo-L-enzymes could be stabilised further by forming CLEAs was examined as follows. First, a zeo-L-enzyme of BglA was prepared using the standard binding assay without the final SDS heat elution step. Then, covalent crosslinks were introduced by treatment with glutaraldehyde to form a zeo-L-enzyme_CLEAs_ (Fig. 2d). Changes in the crosslinker concentration is known to influence CLEA particle size. Therefore, it was expected that CLEA particles would increase in size with increasing concentration of glutaraldehyde as a result of enhanced crosslinking between protein-coated particles. This notion was tested by crosslinking zeo-L-BglA samples with different glutaraldehyde concentrations and comparing the resulting zeo-L-BglA_CLEAs_ particles by scanning electron microscopy (SEM) or field emission SEM (FESEM). Without crosslinking, the particles of the zeo-L-enzymes were well dispersed and less than ~2.5 µm in size (Fig. 3a). Small increases in particle size could be observed after crosslinking with 0.5% glutaraldehyde, (Fig. 3b). At 3% glutaraldehyde (Fig. 3c), large particles ~20 µm in size were produced, and at 10% glutaraldehyde, bulky amorphous particles were obtained with dimensions that exceeded ~40 µm (Fig. 3d). Accordingly, higher glutaraldehyde concentrations initiated a greater degree of crosslinking between the protein coatings of zeo-L-BglA samples, resulting in the formation of larger zeo-L-BglA_CLEAs_.

### Enzymatic activity of zeo-L-enzyme_CLEAs_

**Fig. 4:**
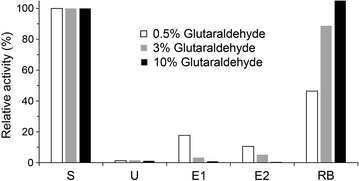
Enzyme activity profile for the immobilisation and subsequent release of L-BglA from zeolite after crosslinking (zeo-L-BglA_CLEAs_) with different concentrations of glutaraldehyde (0.5, 3 and 10%). *S* starting protein, *U* unbound protein, *E1-E2* 1 M betaine elution fractions, *RB* residual bound protein fraction

A successful crosslinking reaction should prevent elution of L-enzymes from the zeolite matrix in the zeo-L-enzyme_CLEAs_ through the strength of the covalent bonds between the enzyme molecules. Various solutions have been formulated for the elution of proteins immobilised onto zeolite via the linker technology [22]. Several of these solutions were tested for their ability to release L-enzymes from zeolite in their active form and 1 M betaine (pH 6) was determined to be the most suitable elution agent (data not shown). The crosslinking reaction was verified by determining the glutaraldehyde concentration required for the effective formation of an enzymatically active zeo-L-enzyme_CLEAs_ and the enzyme activity profile of the resulting zeo-L-enzyme_CLEAs_ was measured after treatment with 1 M betaine. Three different glutaraldehyde concentrations were tested using L-BglA. After the zeo-L-BglA_CLEAs_ were washed with phosphate buffer, 100 µl of 1 M betaine was added and incubated at room temperature for 5 min with rotation. This step was repeated three times to allow for enzyme elution. Standard enzyme activity assays on all fractions and the fractions that displayed activity are shown in Fig. 4. The relative activity of each fraction was calculated as a percentage of the starting material (100%). All the activity of the 10% glutaraldehyde CLEAs was retained in the CLEAs fraction and none was present in the 1 M betaine elution fractions. CLEAs prepared with the lower concentrations of glutaraldehyde, 0.5 and 3%, displayed residual enzyme activities of 55 and 20% less than the 10% glutaraldehyde CLEAs fraction. The remaining activity of the lower glutaraldehyde concentrations was found in the betaine elution fractions. This result indicated that at 0.5 and 3% glutaraldehyde linker-enzymes were not efficiently crosslinked and were consequently eluted from the zeo-L-enzyme_CLEAs_. Although the effective formation of CLEAs by the addition of 0.5 and 3% glutaraldehyde has been reported previously [33], our results showed that these concentrations were insufficient for protein crosslinking and led to enzyme leaching from the CLEAs. In comparison, 10% glutaraldehyde was the most effective concentration, providing a sufficient degree of protein–protein crosslinking that ensured no leaching occurred. Accordingly, 10% (v/v) glutaraldehyde was used for producing CLEAs in all subsequent experiments (including CLEAs from ethanol-precipitated free enzymes).

**Fig. 5:**
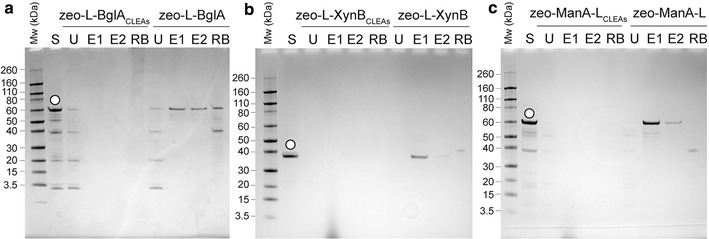
Effect of crosslinking with 10% glutaraldehyde on the immobilisation and release of L-enzymes from zeolite. L-enzymes (*white circles*) were immobilised onto zeolite either with (zeo-L-enzyme_CLEAs_) or without (zeo-L-enzymes) crosslinking; L-enzymes then were released by the addition of 1 M betaine. **a** L-BglA preparations; **b** L-XynB preparations; **c** ManA-L preparations. *S* starting protein, *U* unbound protein, *E1-E2* 1 M betaine elution fractions, *RB* residual bound protein fraction

The process was repeated with L-XynB and ManA-L once 10% was established as the most suitable glutaraldehyde concentration for crosslinking. Activity assays also were performed on all fractions with the resulting zeo-L-XynA_CLEAs_ and zeo-ManA-L_CLEAs_ as described above (results not shown) and protein bands from the different fractions were visualised using SDS-PAGE and Coomassie staining (Fig. 5). Corresponding zeo-L-enzymes (zeolite-bound but not crosslinked) were treated similarly with 1 M betaine elution and used as controls. As shown in Fig. 5, without crosslinking (zeo-L-enzymes), all of the L-enzymes were released from the surface of zeolite upon the addition of the elution agent with the main portion of the released protein located in the first elution fraction (E1) and lesser amounts found in E2. In contrast, the L-enzymes were not eluted from the zeo-L-enzyme_CLEAs_ after betaine treatment with no proteins observed in both the E1 and E2 fractions.

### Characterisation of CLEAs

Both CLEAs and carriers are known to enhance the stability and maintain the activity of enzymes [34–36]. The activities of each thermophilic enzyme were compared as CLEAs prepared from free enzymes without (enzyme_CLEAs_) and with (L-enzyme_CLEAs_) linker and from L-enzymes bound to zeolite (zeo-L-enzyme_CLEAs_).

As shown in Table 3, there was no variation in the optimal pH of the XynB and ManA enzyme_CLEAs_ and L-enzyme_CLEAs_ preparations. However, an increase in pH optimum for activity was observed in BglA prepared similarly. The zeo-L-enzyme_CLEAs_ preparations of L-BglA and L-XynB showed a decrease in their pH optimum when compared to their respective L-enzyme_CLEAs_ whereas the zeo-ManA-L_CLEAs_ showed an increase in pH optimum for activity when compared directly to its L-enzyme_CLEAs_ preparation. The CLEAs preparations of all three enzymes displayed an apparent optimum temperature for activity of 80 °C. In the case of BglA and ManA CLEAs preparations, this is 5 °C below the optimum temperature for activity measured for their respective free enzyme with and without linker (Table 2).

**Table 3:** Effect of forming CLEAs on the optimal pH and temperature of the thermostable enzymes

	BglA	L-BglA	XynB	L-XynB	ManA	ManA-L
Optimum pH						
enzyme_CLEAs_	6.0		6.5		6.0	
L-enzyme_CLEAs_		7.0		6.5		6.0
zeo-L-enzyme_CLEAs_		5.0		6.0		6.5
Optimum temperature (°C)						
enzyme_CLEAs_	80		80		80	
L-enzyme_CLEAs_		80		80		80
zeo-L-enzyme_CLEAs_		80		80		80

### Recycling of immobilised enzymes

**Fig. 6:**
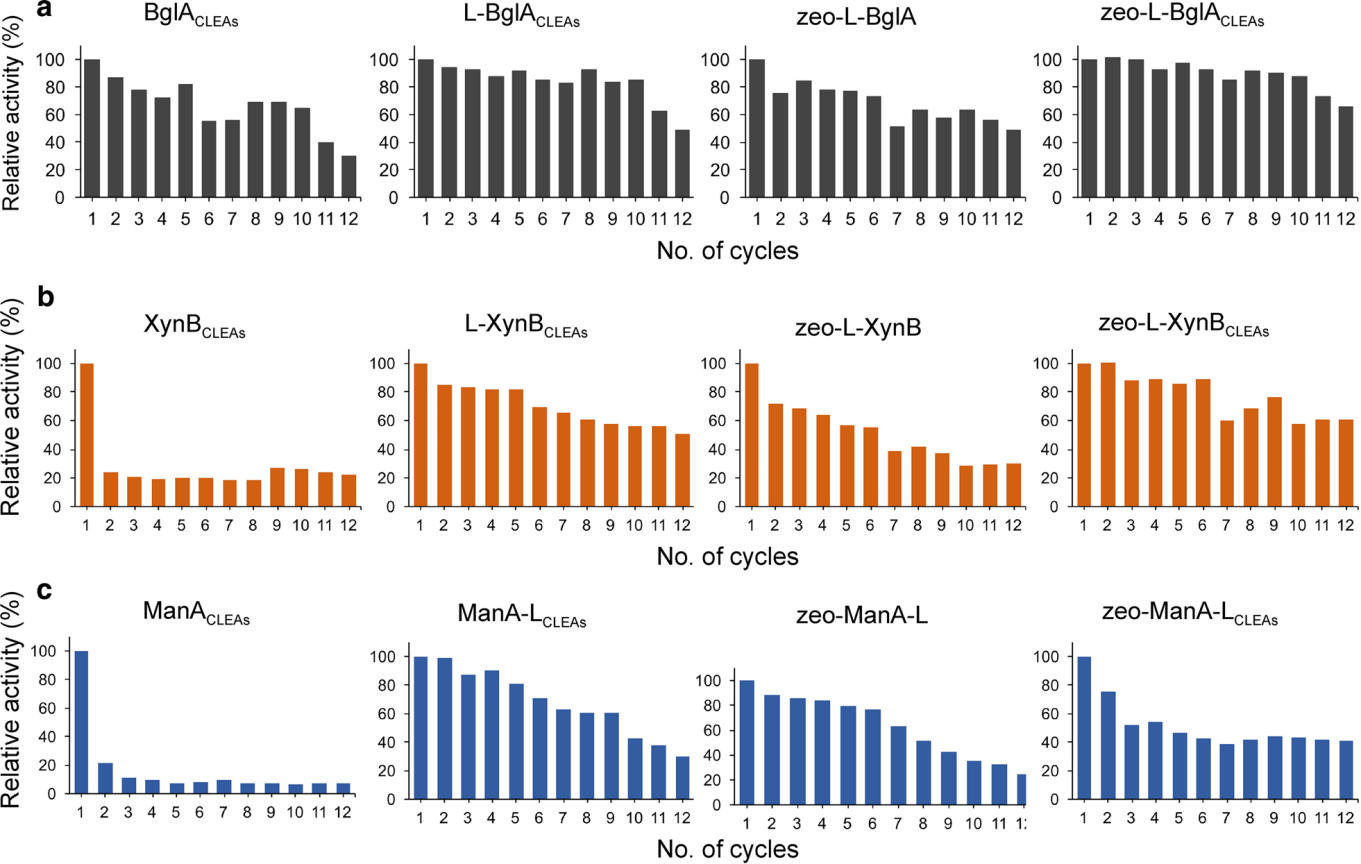
Reusability of each immobilised enzyme preparation over a 12 cycle period. **a** BglA preparations; **b** XynB preparations; **c** ManA preparations. Enzyme activities measurements were performed according to the standard assays at 80 °C for each enzyme

Enzyme immobilisation allows the easy recovery of both enzymes and products, thus significantly reducing overall production costs in biocatalytic processes [31]. The re-usability of the three enzymes was evaluated by using repeated cycles of activity and recovery to determine the degree to which the various CLEAs preparations (enzyme_CLEAs_, L-enzyme_CLEAs_, and zeo-L-enzyme_CLEAs_) and the zeolite-bound enzymes (zeo-L-enzymes) improved activity retention during enzyme recycling (Fig. 6). After 12 cycles of 10 min each, the CLEAs formed from the L-enzymes and zeo-L-enzymes were more resistant to denaturation and retained greater enzyme activity than the enzymes formulated as CLEAs or as zeolite-bound enzymes (zeo-L-enzymes). The zeo-L-BglA_CLEAs_ (Fig. 6a) and zeo-L-XynB_CLEAs_ (Fig. 6b) retained over 60% of their initial activity at the end of the 12 rounds of recycling while zeo-ManA-L_CLEAs_ (Fig. 6c) retained over 40% of its initial activity.

In general, the L-enzyme_CLEAs_ were the next most resistant and stable preparations after the full recycling period with L-BglA_CLEAs_ and L-XynB_CLEAs_ retaining 50% of their initial activity while ManA-L_CLEAs_ retained 30%. In most cases, the CLEAs prepared from the free enzymes without linker (enzyme_CLEAs_) were the least stable preparations with XynB_CLEAs_ and ManA_CLEAs_ losing over 80% of their initial activity after only one incubation cycle. It has been reported previously that the recombinant ManA enzyme exists in both a full and truncated form [20]. Thus, the rapid loss of activity for the ManA_CLEAs_ may be due to an increase in ManA truncation and subsequent release and/or inactivation of the enzyme during recycling. The same CLEAs preparation of BglA (BglA_CLEAs_), on the contrary, was stable for several cycles and still retained 30% of its initial activity after 12 cycles. All of the zeolite-bound enzyme (zeo-L-enzymes) preparations were stable during the recycling period and displayed higher stability profiles than their respective enzyme_CLEAs_ counterparts. The zeo-ManA-L was the next most stable of the ManA preparations, outperforming the zeo-ManA-L_CLEAs_ for the first 8 cycles of the testing period. In general, a combination of linker-mediated zeolite binding and glutaraldehyde crosslinking to form the zeo-L-enzyme_CLEAs_ preparations was the most effective method with which to immobilise these thermostable enzymes as they provided greater enzyme activity retention during the complete recycling period.

### Assembly of single and multiple enzyme biocatalytic modules

Recently, it was shown that CLEAs are able to catalyse a sequence of reactions. These forms of CLEAs have been called combi-CLEAs by some authors [37]. In combi-CLEAs, heterogeneous populations of enzymes can be confined simultaneously in the same aggregate and they perform a versatile cascade or non-cascade bioconversion. For example, thermostable CLEAs of L-arabinose isomerase from *Thermoanaerobacter mathranii* and a β-glycosidase from *Sulfolobus solfataricus* were immobilised by crosslinking with glutaraldehyde and polyethylenimine [38]. In combination, these CLEAs were shown to mediate the two-step conversion of d-galactose to d-tagatose at 65 °C in a single reaction with a 38% conversion.

**Fig. 7:**
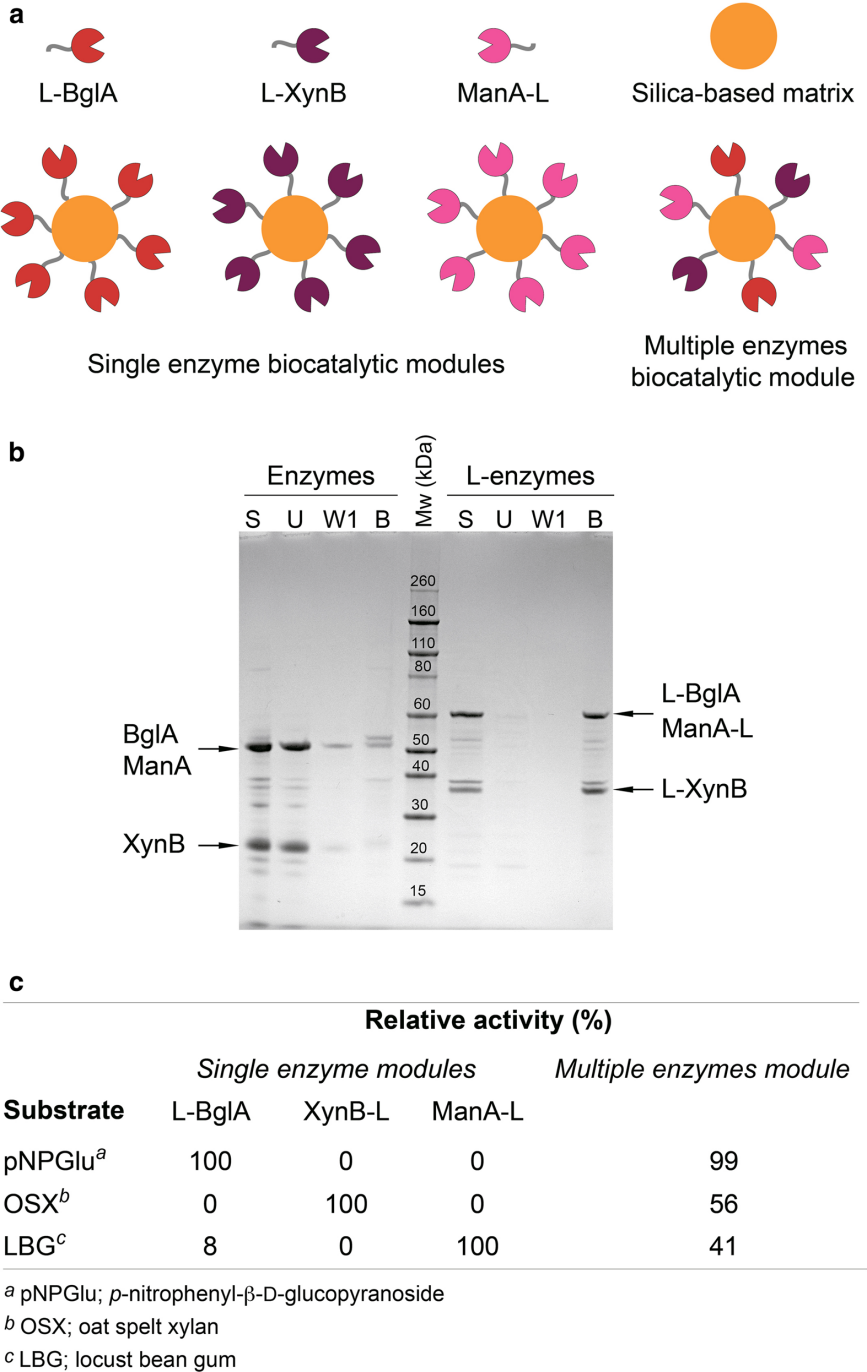
Design, formation and enzymatic activity of biocatalytic modules. **a** Illustration showing the linker-mediated formation of biocatalytic modules consisting of single or multiple types of enzymes; **b** formation of multiple enzyme modules. SDS-PAGE analysis of zeolite binding assays performed with mixtures containing equal amounts of all three enzymes or L-enzymes. *S* starting protein sample, *U* unbound protein fraction, *W1* wash fractions (1 of 3 total), *B* zeolite-bound protein fraction; **c** relative enzymatic activity of single enzyme and multiple enzyme modules against different substrates

Recently, the use of thermostable enzymes immobilised on solid supports has been presented as an alternative approach to produce biocatalytic modules for low-cost biomanufacturing [17, 39, 40]. Biocatalytic modules can be constructed and used to replace industrial chemical catalysts due to their high specificity and high efficiency under mild reaction conditions [17]. In this context, zeo-L-enzyme_CLEAs_ can be considered to be biocatalytic modules with immense potential for industrial applications. The biocatalytic module design relies on the affinity of the linker for low-cost bulk silica-based matrices with additional introduction of crosslinking of the immobilised enzyme. Thus, single biocatalytic modules can be prepared easily consisting of one type of enzyme immobilised per matrix as well as multiple enzyme modules (Fig. 7a).

To demonstrate the potential application of the multiple enzymes module concept, all three enzymes without (BglA, XynB and ManA) and with (L-BglA, L-XynB and ManA-L) the linker were mixed in equal amounts, bound to zeolite using the standard zeolite binding assay and then analysed by SDS-PAGE (Fig. 7b). As expected, all of the L-enzymes exhibited high binding affinity towards zeolite as seen in single binding assays (Fig. 1) with most of the initial protein remaining in the bound fraction. In the zeolite binding assays with the mixed enzymes without the linker, the majority of each of the enzymes was observed in the unbound fraction.

After confirmation that all three mixed L-enzymes were successfully immobilised simultaneously onto zeolite, single and multiple enzyme biocatalytic modules were prepared as described above for zeo-L-enzyme_CLEAs_. Single biocatalytic modules were tested against all three substrates and no module showed significant activity against any substrate except that expected for its constituent enzyme (Fig. 7c). The activity of the multiple enzymes module for each substrate was measured as a percentage of activity of the corresponding single module. The multiple enzymes module showed activity against all three substrates with comparable activity to the single module (L-BglA) against the pNPGlu substrate but only 44 and 59% of the corresponding single module activity against OSX and LBG substrates, respectively.

## Discussion

A silica-binding linker peptide was fused genetically to three thermostable polysaccharide-degrading enzymes for applications in industrial-scale biocatalysis (Table 1). The linker showed high affinity for silica-containing support materials, allowing for directional immobilisation of the three enzymes onto a zeolite matrix (Fig. 1). The enzymes as partially-purified fusion proteins retained both biological activity and binding affinity for zeolite. These results were independent of the position of the linker (N- or C-terminal). The integration of the linker did not have negative effects on the pH optima but lowered the temperature stability of the enzymes (Table 2). SBPs have been shown to have varying effects on the secondary structure of their fusion partners [11]. Hence, a decrease in thermostability may be explained by small changes in the structural conformation of the enzyme after linker fusion, resulting in a loss of thermal stability at high temperatures.

The L-enzymes in several conformations, including being bound to zeolite, were crosslinked individually with glutaraldehyde to form CLEAs and their general properties and activities were essentially unchanged in all cases (Fig. 2; Table 3). For example, protein was not seen in the 1 M betaine elution fractions of the zeo-L-enzyme_CLEAs_, indicating that the crosslinking was effective and no enzyme leakage was observed. The absence of residual bound protein in the SDS-PAGE visualisation of the zeo-L-enzyme_CLEAs_ can be attributed to SDS at high temperatures failing to elute the enzymes due to the presence of the covalent bonds. Additionally, it was found that 0.5 and 3% glutaraldehyde concentrations were not sufficient for the crosslinking reaction to proceed to completion, as evidenced by the activity seen in the elution fractions in Fig. 5. However, our optimisation showed that 10% glutaraldehyde was the most effective concentration for immobilisation.

Previous research comparing CLEAs to native enzymes has shown that enzyme crosslinking broadens the pH values at which the enzyme is active, as well as increasing total activity in U/g/ml. CLEAs also were shown to increase the temperature range at which the enzyme was active [41]. The broadening of activity ranges seen in CLEAs (Table 3) was due to the stabilising nature of the crosslinking reaction. When the enzymes were crosslinked, they were in their native form and the covalent bonds of the crosslinking reaction protected the enzyme from any tertiary structural distortion caused by heat. This effect was shown further by the lack of optimum temperature broadening seen in the free enzymes between the linker and non-linker enzymes, indicating that it was the crosslinking and not the presence of the linker that was responsible for the increased activity range at sub-optimal pH and temperature conditions.

CLEAs made using enzymes immobilised prior to crosslinking display higher activity than both free enzymes and CLEAs made from precipitated free enzymes (Fig. 7) [42]. Research on xylanolytic enzymes immobilised onto silanised magnetic particles and crosslinked showed that the magnetic CLEAs displayed superior thermostability and activity in comparison to both free xylanase and precipitated free enzyme CLEAs.

An advantage of using the enzymes as CLEAs is that it avoids expensive solid supports. However, the linker enables immobilisation onto inexpensive bulk silica-based materials as well as allowing additional properties to be added to the support, for example by employing magnetic silica beads. The linker also adds additional Lys residues for amine-reactive crosslinking and thus provides a benefit to proteins possessing a low Lys content for the crosslinking reaction. It provides directionality for immobilisation so that the individual enzyme molecules are uniformly orientated rather than being bound randomly [43] and eliminates the need for protein precipitants as in the example of zeo-L-enzyme_CLEAs._

In this work, three thermostable glycoside-hydrolysing enzymes were selected as a model system because there is sufficient published data to allow us to compare the performance of our system against studies reported previously [24, 25]. Immobilisation of polysaccharide-degrading enzymes has significant economic and environmental benefits due to its potential for industrial-scale monosaccharide production from minimally-treated plant biomass. Downstream applications include fermentation for cellulosic biofuels, a renewable resource with a much smaller carbon footprint than both traditional petroleum-based fuels and starch-based biofuels [43]. Additionally, the use of cellulosic materials for biofuel production does not compete with food production, unlike starch-based biofuels [44]. The cost of enzymes is one of the most significant expense associated with industrial biocatalysis. Enzyme immobilisation allows the easy recovery of both enzymes and products, multiple reuse of enzymes and the continuous operation of enzymatic processes, thus significantly reducing overall production costs that are frequently a limiting factor in biomass conversion and biocatalytic processes. Enhanced operational performance and the reuse of immobilised enzymes leads to greater catalytic productivity, which reduces the enzyme cost per kg of end product [31]. Large-scale industrial applications require enzyme immobilisation to be simple, without the need for highly purified enzyme preparations or expensive solid supports that limit the commercial viability of the bioprocess. Industrial operations using enzyme immobilisation on inexpensive silica-based supports (e.g. zeolite) combined with enzyme reusage have the capacity to reduce operating and production costs as well as improving the yield of a process.

The zeo-L-enzyme_CLEAs_ of all enzymes display the most activity retention after twelve 10-min cycles, equivalent to 2 h spent at the optimum temperature (Fig. 6). The linker contains 23% Lys or Arg residues, which participate in the crosslinking reaction. The stabilisation provided by these extra residues is a possible reason as to why the zeo-L-enzyme_CLEAs_ are the most stable over 12 recycling assays. The stabilising effect provided by the linker also could provide an explanation as to why the enzyme_CLEAs_ without linkers were the least recyclable of the enzyme preparations. Without the extra stabilisation provided by the linker, the enzyme_CLEAs_ could be more prone to denaturation and loss of activity than immobilised enzymes with linkers. Zeo-L-enzymes display better activity retention than the enzyme_CLEAs_, but showed less recyclability than the L-enzyme_CLEAs_ with and without zeolite. This result implies that the crosslinking reaction plays an important role in stabilising the immobilised enzymes against heat-induced denaturation over subsequent uses. Each of the single zeo-L-enzyme_CLEAs_ showed activity against the substrate of the constituent enzyme and negligible activity towards the other two substrates (Fig. 7c). In contrast, the mixed zeo-L-enzyme_CLEAs_ showed activity towards all three substrates at much lower levels than the single zeo-L-enzyme_CLEAs_ (Fig. 7c).

These results have shown that it is possible for multiple thermostable polysaccharide-degrading enzymes to be immobilised simultaneously onto the same support and retain specific hydrolytic activity. Crosslinking previously immobilised zeo-L-enzymes was the immobilisation method that shows the most potential for industrial applications. The zeo-L-enzyme_CLEAs_ have comparable activity to the free enzymes and superior activity to the enzyme_CLEAs_ with the added ability of being the most recyclable (i.e. retaining the most activity after multiple reactions) and the easiest to remove from the reaction mixture. This fact may have implications in the enzymatic breakdown of lignocellulosic substrates for industrial purposes as well as wider implications in further enzyme immobilisation for production of industrially-useful biomolecules.

In previous studies, cellulolytic enzymes have been immobilised using gold-coated nanoparticles immobilised on thiolated magnetic silica nanoparticles. Cysteine-tagged cellulases were bound to the gold nanoparticles using thiol-gold chemistry [45]. The yields of glucose and cellobiose were found to increase by 179 and 158%, respectively, when using immobilised cellulases compared to the free ones. Stability and activity of the immobilised cellulases remained after four reuses, while the free enzymes dropped to 40% of their previous activity after a single reuse. Plasma immersion ion implantation (PIII) is a technique that reduces the time and amount of steps compared to conventional covalent immobilisation techniques. PIII involves extracting ions from plasma by applying a high voltage direct current which directs the ions to a polymeric substrate covered by a semiconductor wafer. The ions break bonds in the polymer chains and create free radicals that react covalently with the enzyme [46]. However, PIII treatment must be done under vacuum conditions and plasma is created by superheating gas or applying the gas in a strong electromagnetic field. Flat PIII-treated polystyrene was used as a support to immobilise a thermostable β-glucosidase and a commercially-available β-glucosidase. It was found that the thermostable β-glucosidase displayed higher activity when immobilised onto the PIII-treated polystyrene than when adsorbed onto untreated polystyrene [47]. The immobilised thermophilic β-glucosidase also showed 20 times higher activity than the immobilised commercially-available β-glucosidase. PIII has also been used to immobilise a thermophilic β-glucosidase on a curved surface. Using a plastic polymer granules support treated with PIII, a thermophilic β-glucosidase from *Caldicellulosiruptor saccharolyticus* was immobilised and retained activity for 2 weeks, compared to 6 days with untreated granules [48].

The immobilisation technologies described above are elegant and ideally suited for laboratory experiments but do not translate well into industrial-scale processes. Our system, based on low-cost bulk matrices and a simple immobilisation technology, is a viable alternative that requires further investigation and optimisation before application at an industrial scale. Further downstream applications of immobilised polysaccharide-degrading enzymes using this platform technology are in the industrial production of glucose from cellulose in minimally-treated plant biomass. The results reported here indicate that it is possible to set up a degradative pathway for biomass to produce fermentable sugars using inexpensive reagents and matrices that allow the reuse of the enzymatic component at high temperatures and over a number of cycles. In future studies, biocatalytic modules will be designed and constructed for the cell-free assembly of natural and non-natural pathways for the efficient degradation and conversion of biomass into higher-value products.

## Conclusions

The linker technology (and potentially, other solid-binding peptides) represents a novel and inexpensive immobilisation strategy for industrially-significant enzymes. The linker sequence has the capacity to impart directionality and orientation to the immobilisation of enzymes and allows enzyme immobilisation onto cheap bulk matrices for example, zeolite and silica. It provides highly accessible lysine/arginine residues (free amino groups) making it amenable to crosslinking reactions (such as glutaraldehyde) to form CLEAs. Furthermore, the linker system minimises the time spent selecting precipitants and crosslinking reagents. The structural and compositional properties of the linker allow it to impart directionality and orientation to the enzymes after crosslinking (L-enzyme_CLEAs_) resulting in enhanced enzyme reusability. It may be instrumental in allowing crosslinking in a protein–protein only context to form a protein layer around zeolite particles that can enhance thermostability and enzyme activity. The L-enzyme CLEAs lend themselves to facile pathway formation and immobilisation for the optimal hydrolysis of diverse lignin-based biomass sources.

## Methods

### Construction of linker-enzymes

The gene encoding *Caldicellulosiruptor saccharolyticus* β-glucosidase A (*bglA*) was amplified with flanking restriction sites (*Bam*HI and *Hin*dIII) from pProEXTHc-cKanBglA [26] by polymerase chain reaction (PCR) using the primers (forward, 5′-aaaaGGATCCGAGTTTCCCAAAAGGA-3′; reverse, 5′-TTTAATCTGTATCAGGCTGA-3′) and ligated into pET22b (Novagen) plasmids with and without an N-terminal linker sequence [16] to give the expression plasmids pET22-bglA and pET22-L-bglA, respectively.

### Transformation of *Escherichia coli* competent cells

The expression plasmids pET22-L-bglA and pET22-L-xynB contained the linker sequence (L) genetically fused to the N-terminus of *Caldicellulosiruptor saccharolyticus* β-glucosidase A (*bglA*) and *Dictyoglomus thermophilum* β-xylanase B (*xynB*), respectively. The pET22-ManA-L expression plasmid contained the linker sequence fused to the C-terminus of *D. thermophilum* β-mannanase A (*manA*) [20]. The expression plasmids pet22-bglA, pJLA602-xynB [24], and pET22-manA [20] contained each enzyme without the linker sequence. All recombinant constructions and expression hosts are represented diagrammatically in Table 1.

### Recombinant protein expression

*Escherichia coli* Tuner (DE3) or BL21 harbouring the expression plasmid of interest were cultured in Luria-Bertani (LB) medium supplemented with 50 µg/ml carbenicillin for the production of all recombinant enzymes with and without the linker. Cells were grown at 37 °C with shaking (250 rpm) until the A_600_ was between 0.7 and 0.9. Recombinant protein synthesis (except for XynB) was induced by the addition of 0.4 mM isopropyl β-d-thiogalactoside (IPTG). The expression plasmid pJLA602-XynB is heat-inducible and requires an increase in growth temperature to 42 °C to initiate the expression of XynB [49]. After a further 3–4 h of cultivation at 37 °C (42 °C for XynB), cells were harvested by centrifugation for 15 min at 4000×*g* at 4 °C and stored at −20 °C.

Cells were resuspended in ice-cold lysis buffer (25 mM Tris–HCl, pH 8.0, 100 mM NaCl, 1.25 mM EDTA and 0.05% Tween 20) supplemented with 4 mM of the serine protease inhibitor Pefabloc (Sigma) and 1.5 mg lysozyme (Sigma). Cells were disrupted by three passages through a French pressure cell and then 25 U Benzonase (Novagen) and a further 4 mM of Pefabloc were added. Cellular debris was removed by centrifugation for 30 min at 4000×*g* at 4 °C following incubation on ice for 20 min. The supernatant obtained was incubated for 30 min at 70 °C to denature host proteins and to partially purify the thermostable recombinant protein. Heat-denatured proteins were removed by centrifugation at 4000×*g* for 30 min at 4 °C. The supernatant obtained was filtered through a 0.2 µm sterile filter (Millipore), supplemented with 0.05% sodium azide and stored at 4 °C. All partially purified protein concentrations were determined by measuring the absorbance of each sample at 280 nm on a Nanodrop 2000c spectrophotometer (Thermo Scientific).

### Standard zeolite binding assay

The synthetic zeolite CBV-100 (Zeolyst International, USA) was used as the silica-based matrix in this study. Standard zeolite binding assays were performed as described previously [16]. Briefly, 5 mg of zeolite was washed three times with 500 µl washing buffer (1% Triton X-100 in 50 mM phosphate buffer, pH 6.0). Soluble, partially purified enzyme (20 µg in 100 µl of phosphate buffer) was mixed with the washed zeolite and incubated with rotation for 30 min at room temperature. After centrifugation at 10,000×*g* for 30 s, the supernatant containing the unbound fraction was removed. The zeolite pellet then was washed three times with 100 µl of 50 mM phosphate buffer (pH 6.0) by vortexing and centrifugation. Zeolite-bound proteins were eluted from the matrix using 100 µl SDS-PAGE loading buffer and incubation at 99 °C for 10 min. Fractions containing protein were separated on NuPage 4–12% Bis–Tris Gels (Life Technologies) by SDS-PAGE and identified with Coomassie Brilliant Blue staining.

### Standard enzyme assays

All reaction mixtures consisted of an appropriate amount of enzyme (20 µl) and their respective substrate (80 µl). Reactions were prepared in triplicate and incubated at 80 °C for 10 min. Each enzyme was diluted to a concentration that was not substrate limited during the assay reaction. In controls, each enzyme was added after the termination of the reaction. For comparison and convenience, all enzyme activities are expressed as a percentage of the maximal activity.

β-Glucosidase activity was assayed using 0.5 mg/ml *p*-nitrophenyl-β-d-glucopyranoside (pNPGluc, MP Biomedicals) substrate in 40 mM universal buffer [50]. The assay reactions were terminated by addition of 100 µl 1 M Na_2_CO_3_ and the absorbance of the released *p*-nitrophenol was measured at 405 nm using a PHERAstar FS 96-well plate reader (BMG Labtech, Germany).

β-Xylanase and β-mannanase activities were assayed using 0.5% (w/v) oat spelt xylan (OSX, Sigma) and locust bean gum (LBG, Sigma) substrates in 120 mM universal buffer, respectively. The reducing sugars released were measured using the dinitrosalicylic acid (DNS) assay procedure [51] and the absorbance increase generated by the release of 3-amino-5-nitrosalicylic acid was measured at 540 nm using a PHERAstar FS 96-well plate reader.

### Activity characterisation of free enzymes

The effects of pH and temperature on the free enzyme activities were examined using the partially purified recombinant proteins. The effect of temperature on the reaction rate was determined by incubating the free enzyme with the appropriate substrate at temperatures ranging from 40 to 90 °C under standard assay conditions. To determine the optimal pH, the appropriate substrate was prepared in universal buffer and assayed at different pH values (all pH values were adjusted at the temperature of the assay) at the optimal temperature for activity. To determine the thermostability of the free enzymes, they were incubated between 70 and 90 °C, in the absence of substrate. Samples were removed at different time intervals and the residual enzyme activity was measured under standard enzyme assay conditions with an assay time of 10 min.

### Enzyme immobilisation

#### Zeolite-bound linker-enzymes (zeo-L-enzyme)

Zeolite-bound linker-enzymes were prepared using the standard zeolite binding assay without the final SDS heat elution step. The resulting final insoluble zeolite-bound fraction was resuspended in 50 mM phosphate buffer (pH 6.0).

#### CLEAs of free enzymes (enzyme_CLEAs_ and linker-enzyme_CLEAs_)

CLEAs of each free enzyme (with and without linker) were prepared by a combined one-step ethanol precipitation and crosslinking strategy. Glutaraldehyde (0.5, 3.0 and 10% v/v) in chilled 100% ethanol to a total volume of 9 ml was added to 1 ml of each of the partially purified free enzymes and incubated overnight at 4 °C without shaking. The enzyme_CLEAs_ formed were centrifuged for 4000×*g* for 30 min at 4 °C, and the supernatant tested for residual enzyme activity. The absence of detectable activity in the supernatant was taken as an indication the aggregation and crosslinking had gone to completion. The enzyme_CLEAs_ were washed three times in 50 mM phosphate buffer (pH 6.0) to remove any unreacted glutaraldehyde.

#### CLEAs of zeolite-bound linker-enzymes (zeo-L-enzyme_CLEAs_)

Single enzyme biocatalytic modules: CLEAs of each zeolite-bound linker-enzyme were prepared using standard zeolite binding assays without the final SDS heat elution step (see Fig. 6a), followed by crosslinking with 100 µl glutaraldehyde (0.5, 3.0 and 10% v/v). After incubation at 4 °C with rotation for 1.5 h, the resulting zeo-L-enzyme_CLEAs_ were washed three times with 500 µl of 50 mM phosphate buffer (pH 6.0) to remove any unreacted glutaraldehyde.

Multiple enzyme biocatalytic modules: A mixed zeo-L-enzyme_CLEAs_ was prepared using an initial combined mixture containing 100 µl of each of the three linker-enzymes in a single zeolite standard binding assay (see Fig. 6a) as described above for Single enzyme biocatalytic modules. The resulting mixed zeo-L-enzyme_CLEAs_ were resuspended in 100 µl 50 mM phosphate buffer (pH 6.0). A 10 µl sample was wet-mounted onto a glass slide for light microscopy to ascertain that the crosslinking reaction had been successful.

### Recycling immobilised enzymes

Recycling assays were performed with the four forms of the immobilised enzymes (enzyme_CLEAs_, L-enzyme_CLEAs_, zeo-L-enzymes and zeo-L-enzyme_CLEAs_). Each enzyme was incubated with its appropriate substrate under standard assay conditions. After an incubation period of 10 min, each reaction was centrifuged briefly to pellet the immobilised enzyme and the supernatant obtained was transferred to a fresh tube for the addition of the stopping reagents DNS or Na_2_CO_3_. Fresh substrate was then added to each immobilised enzyme and the standard assay was repeated. The immobilised enzymes were recycled in this manner for a total of 12 cycles.

### Multiple substrate hydrolysis

Each of the constituent enzymes in the mixed and individual zeo-L-enzyme_CLEAs_ were tested for activity against each substrate (OSX, LBG and pNPGluc) under the standard assay conditions to determine whether the mixed zeo-L-enzyme_CLEAs_ were capable of carrying out their hydrolysis reactions. The relative activity of the mixed zeo-L-enzyme_CLEAs_ against each substrate was calculated as a percentage of the activity of the individual zeo-L-enzyme_CLEAs_ against the same substrate.

### Scanning electron microscopy

Samples of zeo-L-BglA_CLEAs_ (5 µl) were snap frozen and lyophilised overnight onto electron microscopy coverslips and mounted onto adhesive coverslip stands. The samples were gently agitated with air to remove loose particles, after which they were coated in gold using an Emitech K550 Gold Sputter Coater Unit. A JEOL-SM-6480 scanning electron microscope was used to image the samples.

## Abbreviations

AP: alkaline phosphatase
BglA: β-glucosidase
CLEAs: cross-linked enzyme aggregates
DNS: dinitrosalicylic acid
EDTA: ethylenediaminetetraacetic acid
enzyme_CLEAs_: CLEAs of enzymes without the linker
FESEM: field emission scanning electron microscopy
GBP: gold-binding peptide
IPTG: isopropyl β-d-thiogalactoside
L: linker
LB: Luria–Bertani medium
L-enzymes: enzymes with the linker
L-enzyme_CLEAs_: CLEAs of L-enzymes
LBG: locust bean gum
ManA: β-mannanase
OSX: oat spelt xylan
PIII: plasma immersion ion implantation
pNPGlu: *p*-Nitrophenyl-β-d-glucopyranoside
SBP: solid-binding peptide
SDS-PAGE: sodium dodecyl sulfate polyacrylamide gel electrophoresis
SEM: scanning electron microscopy
zeo-L-enzymes: zeolite-bound L-enzymes
zeo-L-enzyme_CLEAs_: CLEAs of zeolite-bound L enzymes
XynB: β-xylanase
